# Association of aspirin use alone with mortality and liver-related events in MASLD: a multi-institutional three-year study

**DOI:** 10.1080/07853890.2025.2573146

**Published:** 2025-10-17

**Authors:** Chien-Hao Huang, Chun-Li Wang, Victor Chien-Chia Wu, Yi-Chung Hsieh, Chia-Ling Wu, Z-Fan Zeng, Yu-Tung Huang, Chao-Wei Hsu, Rong-Nan Chien, Shang-Hung Chang

**Affiliations:** ^a^ Division of Hepatology, Department of Gastroenterology and Hepatology, Chang-Gung Memorial Hospital, Linkou Medical Center, Linkou, Taiwan; ^b^ College of Medicine, Chang-Gung University, Taoyuan City, Taiwan; ^c^ Liver Research Center, Chang Gung Memorial Hospital, Linkou, Taiwan; ^d^ Department of Cardiology, Chang-Gung Memorial Hospital, Linkou Medical Center, Taoyuan City, Taiwan; ^e^ Department of Medical Research and Development, Center for Big Data Analytics and Statistics, Chang Gung Memorial Hospital, Linkou Medical Center, Taoyuan City, Taiwan

**Keywords:** Metabolic dysfunction-associated steatotic liver disease (MASLD), non-alcoholic fatty liver disease (NAFLD), hepatocellular carcinoma (HCC), liver-related mortality, all-cause mortality, major bleeding events, antiplatelet therapy

## Abstract

**Background:**

The global prevalence of metabolic dysfunction-associated steatotic liver disease (MASLD), previously known as NAFLD, is increasing. While daily aspirin use has been associated with reduced hepatocellular carcinoma (HCC) risk in NAFLD, its impact on mortality and liver-related outcomes in MASLD remains unclear.

**Methods:**

This retrospective, multi-institutional cohort study included 48,722 MASLD patients from 2008 to 2020, divided into aspirin users and non-users. Exclusion criteria included significant cardiac disease unrelated to aspirin, alcoholic liver disease, incomplete follow-up, and concomitant use of other antiplatelet therapies. Propensity score matching (PSM) was performed to balance baseline characteristics, including demographics, cirrhosis status, ALT levels, cardiovascular conditions, and concurrent medications.

**Results:**

After PSM, 2003 patients were included in each cohort. Aspirin users had a mean duration of use of 4.59 ± 3.97 years, with 97% on a daily dose of 75–100 mg. Over three years, aspirin use was not significantly associated with reductions in overall mortality, liver-related events, liver-related mortality, or HCC incidence compared to non-users. However, a trend toward lower liver-related mortality and HCC incidence was observed without an increased risk of major bleeding.

**Conclusion:**

In this large cohort, daily aspirin use alone did not significantly reduce mortality or liver-related events in MASLD patients over three years. The observed trends may suggest potential long-term benefits, warranting further investigation into extended aspirin duration and combination strategies for MASLD management.

## Introduction

The global burden of metabolic dysfunction-associated steatotic liver disease (MASLD), formerly termed non-alcoholic fatty liver disease (NAFLD), has become a significant public health challenge. In 2023, a multi-society Delphi consensus recommended the term ‘MASLD’ to better emphasize its metabolic underpinnings and its close associations with obesity, diabetes, and dyslipidemia [1,2]. This change reflects the complexity of MASLD’s progression, which can lead to advanced fibrosis, cirrhosis, and ultimately hepatocellular carcinoma (HCC) [3–5]. Notably, unlike NAFLD, MASLD is not mutually exclusive with viral hepatitis, as metabolic dysfunction can coexist with chronic viral liver disease [6].

Although several investigational drugs for MASLD have shown promise in clinical trials, obtaining FDA approval remains challenging [7]. Notably, Resmetirom, a thyroid hormone receptor-β agonist, has been approved for non-alcoholic steatohepatitis (NASH) patients with moderate to advanced liver fibrosis due to its efficacy in improving steatohepatitis and fibrosis [8,9]. Its long-term impact on mortality and liver-related events, including HCC, remains under investigation.

Aspirin, widely recognized for its anti-inflammatory properties, has been extensively studied for its chemopreventive effects in inflammation-driven cancers, particularly colorectal cancer [10,11]. In patients with chronic hepatitis B or C, long-term aspirin use has been more definitively associated with a reduced incidence of HCC [12–15]. However, its efficacy in MASLD remains inconclusive. Experimental and animal studies suggest that antiplatelet therapy, such as aspirin, may attenuate fibrosis progression in biopsy-confirmed NAFLD patients [16,17], potentially lowering the risk of NAFLD-related HCC [18] and even reducing liver-related mortality [19]. Furthermore, a recent preliminary randomized clinical trial reported that six months of daily low-dose aspirin noticeably reduced hepatic fat content in MASLD patients compared to the placebo [20].

Despite these promising preliminary findings, the long-term impact of daily aspirin use on overall mortality, liver-related events, liver-related mortality, and HCC incidence in MASLD patients remains uncertain, particularly as previous studies have often included patients with a range of cardiac comorbidities [18], potentially affecting outcomes and introduces significant confounding factors, making it difficult to isolate aspirin’s direct hepatic effects from its cardiovascular benefits. Further previous studies often include concurrent use of other antiplatelet agents, given the true effect of aspirin alone less defined. Additionally, the benefit-risk balance of long-term aspirin use (≥5 years) in individuals without significant thromboembolic risk remains unclear and warrants careful consideration.

In this study, we assess the impact of daily aspirin on these outcomes over a three-year mid-term period in MASLD patients. By selecting well-balanced cohorts, isolating aspirin exposure, and applying rigorous statistical methods to mitigate immortal time bias, we aim to provide refined insights that build upon and enhance prior research.

## Materials and methods

### Data source

Data were retrieved from the electronic medical records (EMR) of the multi-institutional Chang Gung Memorial Hospital system to investigate the therapeutic effect of aspirin on MASLD patients. The hospital system spans from northern to southern Taiwan and includes three tertiary care medical centers and four teaching hospitals. It provides medical care to more than 6.1% (>1.3 million) of outpatients and 10.2% (>0.2 million) of inpatients annually in Taiwan [21]. These data include outpatient, emergency, and inpatient claim records, diagnoses, prescriptions, laboratory examinations, operations, and records of imaging, ultrasonography, endoscopy, microbiology, and pathology reports [22].

### Study design and patient cohorts

**Figure 1.**
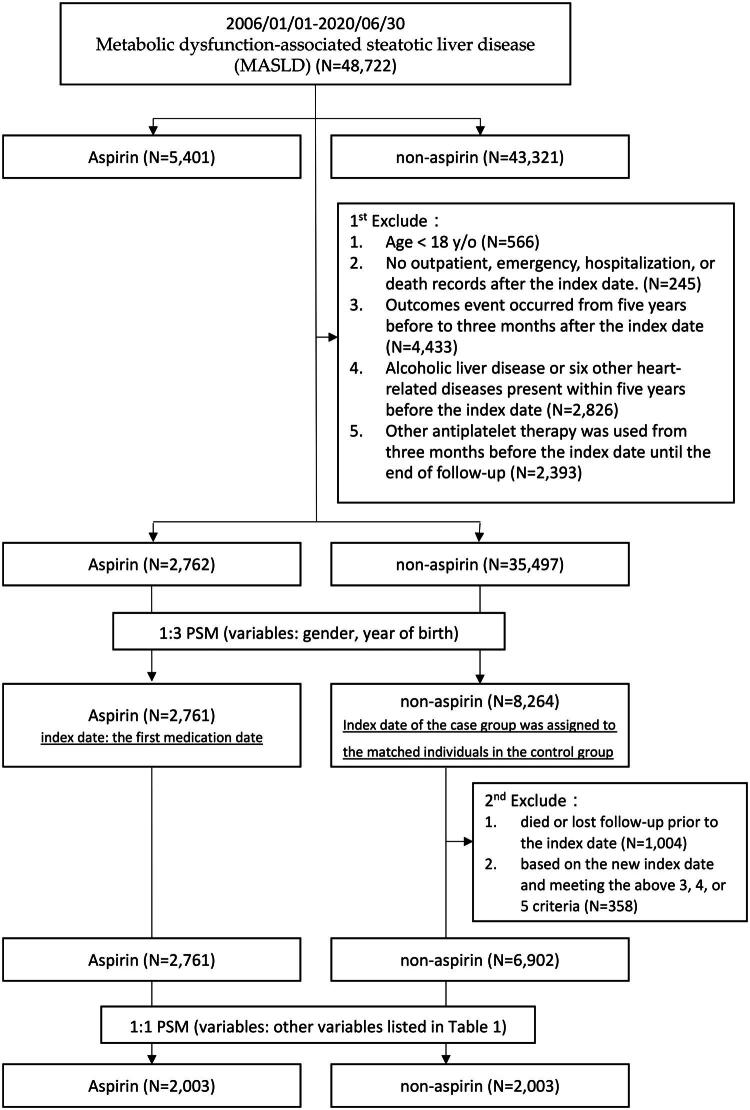
Flowchart of the study.

This retrospective cohort analysis incorporated a substantial real-world MASLD patient cohort from 1 January 2006 to 30 June 2020. Patients were identified using the International Classification of Diseases, Ninth Revision (ICD-9) codes 571.8, 571.9, and the Tenth Revision (ICD-10) codes K76.0 and K75.81. The diagnosis was confirmed through abdominal ultrasonography or histopathological evidence. Inclusion criteria required patients to meet at least one of the five cardiometabolic criteria defined by the MASLD Delphi consensus (e.g. BMI, blood pressure, laboratory data) [1] at the index date. Patients with coexisting viral hepatitis were also included [18]. A detailed patient selection flowchart is illustrated in Figure 1.

Subsequently, exclusions were applied to the following individuals: those younger than 18 years, and patients without follow-up data on outpatient visits, emergency room (ER) visits, hospital admissions, or mortality records after the index date. Additionally, patients with any outcome events- such as a history of hepatocellular carcinoma (HCC), liver-related events, all-cause and liver-related mortalities, composite CVD events (ischemic stroke, myocardial infarction, heart failure, and cardiovascular death) [23]—occurring within five years before or three months after the index date were excluded. Patients with significant cardiac disease unrelated to aspirin—including those with a history of heart failure, arrhythmias, structural heart disease requiring medications other than aspirin, or any history of six major cardiovascular conditions unrelated to the composite CVD events (aortic dissection, pericarditis, endocarditis, myocarditis, rheumatic heart disease, and pulmonary hypertension, as defined in Supplementary Table 1) within five years before the index date—were excluded to minimize confounding and focus on MASLD-related outcomes. Additionally, patients diagnosed with alcoholic liver disease, based on ICD codes for alcohol dependence or abuse (Supplementary Table 1), were excluded. Lastly, patients using other antiplatelet therapies for 84 days or more from three months before the index date until the end of follow-up were excluded from the analysis.

After applying the five major exclusion criteria, the remaining patients were stratified into two groups: an aspirin cohort and a non-aspirin cohort. In the aspirin cohort, the index date was defined as the date of the first aspirin prescription, and patients must have taken aspirin for a minimum of 84 consecutive days to be included. To address immortal time bias in the non-aspirin cohort, a series of steps were undertaken (Figure 1): (1) patients in the aspirin group were matched 1:3 with non-aspirin group patients by gender and birth year using propensity-score matching (PSM); (2) the index dates for aspirin cohort patients were assigned to the corresponding non-aspirin cohort patients; (3) patients in the non-aspirin group who died, were lost to follow-up, or met exclusion criteria 3, 4, and 5 prior to the index date were excluded; and (4) finally, a 1:1 PSM was conducted to ensure comparability between the two cohorts, adjusting for key baseline variables, including demographic data, prevalence of liver cirrhosis, elevated serum alanine aminotransferase (ALT) levels (>36 U/L), cardiovascular comorbidities (diabetes, hyperlipidemia, hypertension, coronary artery disease, hemorrhagic stroke, cardiac arrhythmia, and peripheral vascular disease), use of common MASLD-related co-medications (e.g. metformin-containing oral hypoglycemic agents, statins, proton pump inhibitors, histamine-2 receptor antagonists), and viral etiology. PPIs and H2RAs were included specifically because of their widespread use in patients with advanced liver disease and their potential impact on the gut–liver axis, including associations with bacterial translocation and altered microbiota, which may influence liver-related complications [24].

### Study outcomes and follow-ups

The primary outcomes of this study were all-cause mortality and liver-related events at least three months after the index date. Liver-related events were defined as the occurrence of esophageal variceal bleeding, ascites, hepatic encephalopathy, spontaneous bacterial peritonitis, liver transplantation, hepatorenal syndrome (for detailed definitions, refer to Supplementary Table 2) [18,25].

The secondary outcomes were the development of HCC and liver-related mortality, while the safety outcome focused on major bleeding events, defined according to the criteria established by the International Society on Thrombosis and Haemostasis (ISTH). These criteria included intracranial bleeding, gastrointestinal bleeding, and other critical site bleeding [26], with detailed descriptions all provided in Supplementary Table 3. To assess steatosis and fibrosis, common non-invasive diagnostic tools were utilized at both the index date and three years post-index, such as the NAFLD-Liver Fat Score [27,28], Hepatic Steatosis Index (HSI) [29], NAFLD Fibrosis Score [30], BARD score [31], AST to Platelet Ratio Index (APRI), and Fibrosis-4 (FIB-4). The delta changes in these scores over the three years were compared between the aspirin-treated cohort and the control cohort.

Patients were regularly followed, and outcomes were assessed until the occurrence of a predefined outcome event, death, or the completion of a 3-year follow-up period, whichever occurred first.

### Statistical analysis

Descriptive statistics were used to summarize continuous variables as mean ± standard deviation (SD) for normally distributed variables or as median ± interquartile range (IQR) for non-normally distributed variables, as assessed using graphical methods (e.g. histograms) and numerical tests (e.g. skewness, kurtosis). Categorical variables were summarized as frequencies and percentages. To ensure comparability between the case and control cohorts, key baseline factors—including age, sex, the prevalence of liver cirrhosis, elevated serum ALT levels, cardiovascular comorbidities, and the use of co-medications (e.g. metformin-containing oral hypoglycemic agents, statins, proton pump inhibitors (PPI), histamine-2 receptor antagonists (H2RA), and antiplatelet therapies)—were adjusted using 1:1 PSM. An absolute standardized mean difference (ASMD) < 0.1 indicated no significant difference between the groups post-PSM.

Competing risk analyses were performed to assess the subdistribution hazard ratio (SHR) of the primary and secondary outcomes over a 3-year follow-up. The magnitude of delta changes in various steatosis and fibrosis scores pre- and 3 years post the index date, as presented in Supplementary Table 5, were compared using either independent t-tests or Wilcoxon rank-sum tests, depending on the normality of the data as assessed by Shapiro-Wilk tests. Non-parametric methods were selected when normality assumptions were not met or when follow-up data were limited for specific NITs. A two-sided p-value of <0.05 was considered statistically significant. All statistical analyses were performed using SAS software, version 9.4 (SAS Institute, Cary, NC, USA).

### Ethics statement

This study was conducted following the Declaration of Helsinki and the Declaration of Taipei regarding ethical considerations for health databases by the World Medical Association. The study protocol was approved, and the need for informed consent was waived by the Institutional Review Board of Chang Gung Medical Foundation (IRB No: 202101529B0). Exemption was granted as all data, converted from the database claims records, were anonymized to comply with the stringent regulations for on-site analysis at CGMH’s big data center. To ensure patient confidentiality, all patient identification numbers were encrypted and deidentified.

## Results

### Demographics

Between 1st January 2006 and 30th June 2020, a total of 48,722 patients (5401 aspirin users, 43,321 non-aspirin users) met the MASLD definition based on the 2023 consensus by fulfilling the five cardiometabolic criteria assessed at baseline (index date) (Figure 1). Following the 1^st^ exclusion process, 38,259 patients (2762 aspirin users, 35,497 non-aspirin users) were selected for further analysis. To address immortal time bias, a 1:3 PSM was performed, followed by the 2^nd^ exclusion, leaving 2761 aspirin users and 8264 non-aspirin users. To ensure comparability between cohorts, a 2^nd^ PSM was conducted Ultimately, 2003 patients from both the aspirin and non-aspirin cohorts were matched in a 1:1 ratio for detailed comparative analysis.

In Table 1, the baseline clinical characteristics-including non-invasive liver fibrosis tests-are compared between the aspirin cohort and the non-aspirin cohort. All variables exhibited an ASMD< 0.1, signifying a high degree of baseline comparability. Notably, within the aspirin cohort, 97.10% of patients were prescribed ≤100 mg/day of aspirin while 2.90% received doses > 100 mg/day. The mean duration of aspirin therapy was recorded at 4.59 ± 3.97 years.

**Table 1.** Comparison of baseline clinical characteristics between propensity score-matched MASLD patient cohorts who took aspirin ≧ 84days vs. Without aspirin (1:1).

Baseline Clinical Characteristics @	Case (Aspirin-treated) (*n* = 2003)	Control (No aspirin-treated) (*n* = 2003)	ASMD^#^
Age (years), mean ± SD	63.63 ± 11.81	63.87 ± 11.22	0.021
Sex, n (%)			0.017
Male	1206 (60.21)	1189 (59.36)	
Female	797 (39.79)	814 (40.64)	
Aspirin dosage, n (%)			N/A
≤100 mg/day	1945 (97.10)		
>100 mg/day	58 (2.9)		
Aspirin duration, year			N/A
Mean ± SD	4.59 ± 3.97		
Liver Cirrhosis, n (%)	32 (1.6)	38 (1.9)	0.023
Viral etiology, n (%)			
HBV	249 (12.43)	277 (13.83)	0.041
HCV	93 (4.64)	84 (4.19)	0.022
HBV+HCV	18 (0.90)	20 (1.00)	0.010
ALT elevation, n (%)	819 (40.89)	828 (41.34)	0.009
CV-related disease, n (%)			
Diabetes	1085 (54.17)	1128 (56.32)	0.043
Hyperlipidemia	1582 (78.98)	1583 (79.03)	0.001
Hypertension	297 (14.83)	55 (12.73)	0.061
CAD	231 (11.53)	229 (11.43)	0.003
Hemorrhagic stroke and/or miscellaneous	193 (9.46)	190 (9.49)	0.004
Cardiac arrhythmia	180 (8.89)	178 (8.89)	0.004
PVD	50 (2.50)	52 (2.60)	0.006
Cardiomyopathy	4 (0.2)	6 (0.3)	0.020
Non-rheumatic heart dz.	42 (2.1)	43 (2.15)	0.003
Drug use, n (%)			
Metformin*	635 (31.70)	628 (31.35)	0.008
Statin	845 (42.19)	840 (41.94)	0.005
PPI	115 (5.74)	141 (7.04)	0.053
H2RA	108 (5.39)	124 (6.19)	0.034
Non-invasive tests(fibrosis)^$^			
FIB-4 index(n = 165)	4.06 ± 5.80	3.54 ± 4.33	0.068
APRI (n = 168)	1.13 ± 2.78	1.07 ± 1.36	0.028
MELD score (n = 645)	8.51 ± 1.22	8.63 ± 1.21	0.099

@: values are expressed in n (%) or mean ± standard deviation; ASMD **^#^**: absolute standardized mean difference. SMD < 0.1 means no significant difference between the two groups. *Metformin-containing oral hypoglycemic agent. **^$^**Not included in the PSM and only for descriptive purposes and post-PSM balance assessment. N/A = not applicable, SD = standard deviation, ALT = alanine aminotransferase, CAD = coronary arterial disease, CV = cardiovascular, CVD = cerebral vascular disease, PVD = peripheral vascular disease, PPI = proton pump inhibitor, H2RA = histamine 2 receptor antagonist, APT = antiplatelet; HBV: chronic hepatitis B viral infection; HCV: chronic hepatitis C viral infection, FIB-4 index = Fibrosis-4 index, APRI: AST to Platelet Ratio Index; MELD = The Model for End-Stage Liver Disease.

### Primary outcomes

**Figure 2.**
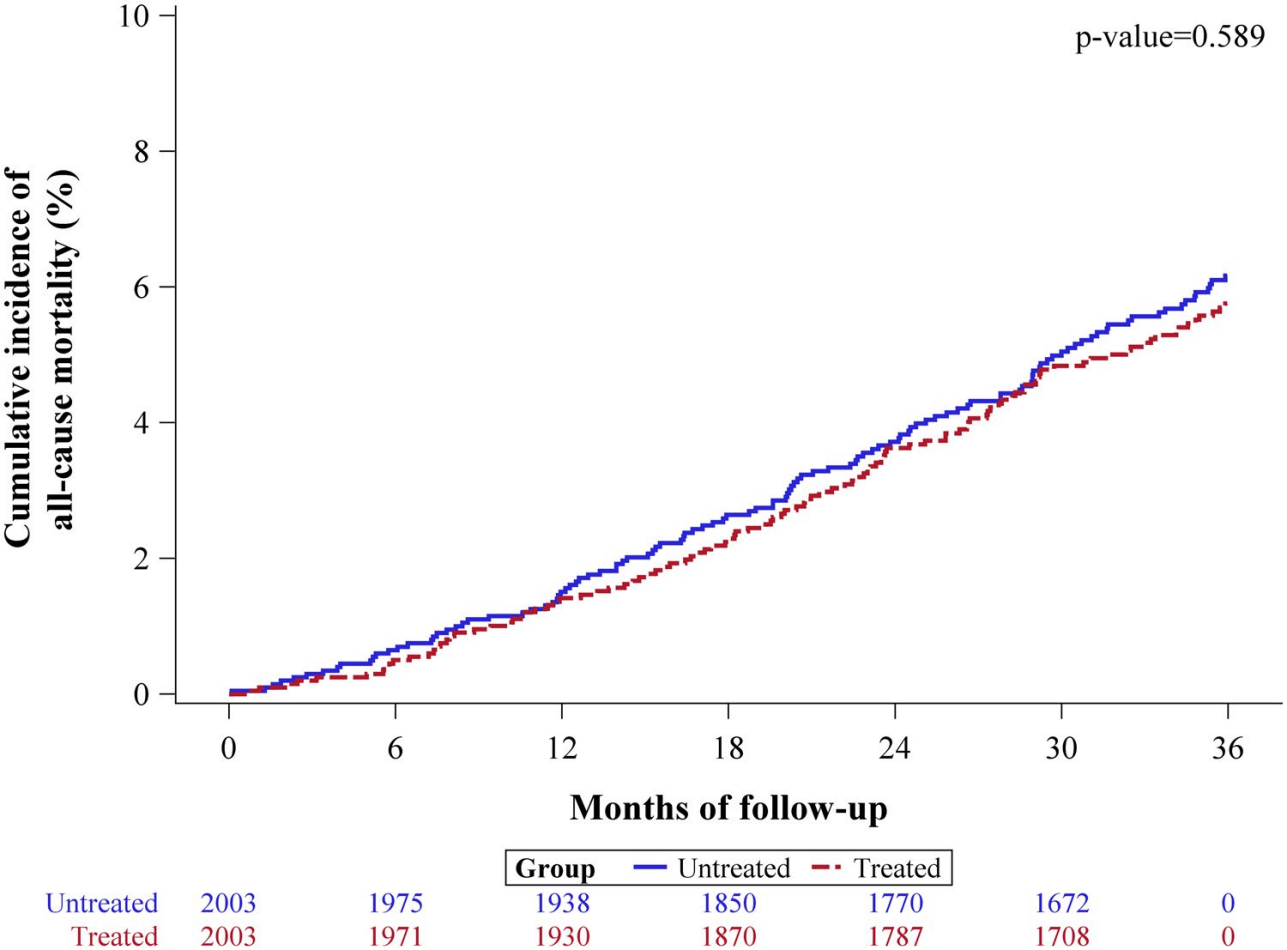
Cumulative Incidence Function (CIF) plot for all-cause mortality over a three-year follow-up period after adjustment for immortal time bias.

A competing risk analysis was conducted to assess the independent effect of aspirin use on all-cause mortality, considering liver transplantation (LT) as a competing event. Among 4,006 patients (aspirin: 2,003; non-aspirin: 2,003) included in the PSM cohort, 108 patients (5.4%) in the aspirin cohort and 115 patients (5.7%) in the non-aspirin cohort experienced all-cause mortality during the three-year follow-up period. The analysis revealed no significant reduction in the SHR for all-cause mortality in the aspirin cohort (SHR: 0.93, 95% CI: 0.72–1.21, *p* = 0.559) (Table 2 and Figure 2).

**Table 2.** Competing risk analysis for the primary and secondary clinical outcomes over 3 years of follow-up.

Clinical outcome	N	Events	SHR	95%CI	*p*-value	Competing risk
All-cause mortality						LT
Aspirin	2003	108	0.93	(0.72–1.21)	0.5589	
Non-aspirin	2003	115				
Liver- related events						
Aspirin	2003	30	0.88	(0.54–1.43)	0.596	All-cause mortality
Non-aspirin	2003	34				
Liver-related mortality						
Aspirin	2003	7	0.49	(0.20–1.22)	0.126	LT
Non-aspirin	2003	14				
HCC						LT, all-cause mortality
Aspirin	2003	14	0.63	(0.32–1.23)	0.178	
Non-aspirin	2003	22				

N, number of patients; SHR, subdistribution hazard ratio; CI, confidence interval; LT, liver transplantation.

**Figure 3.**
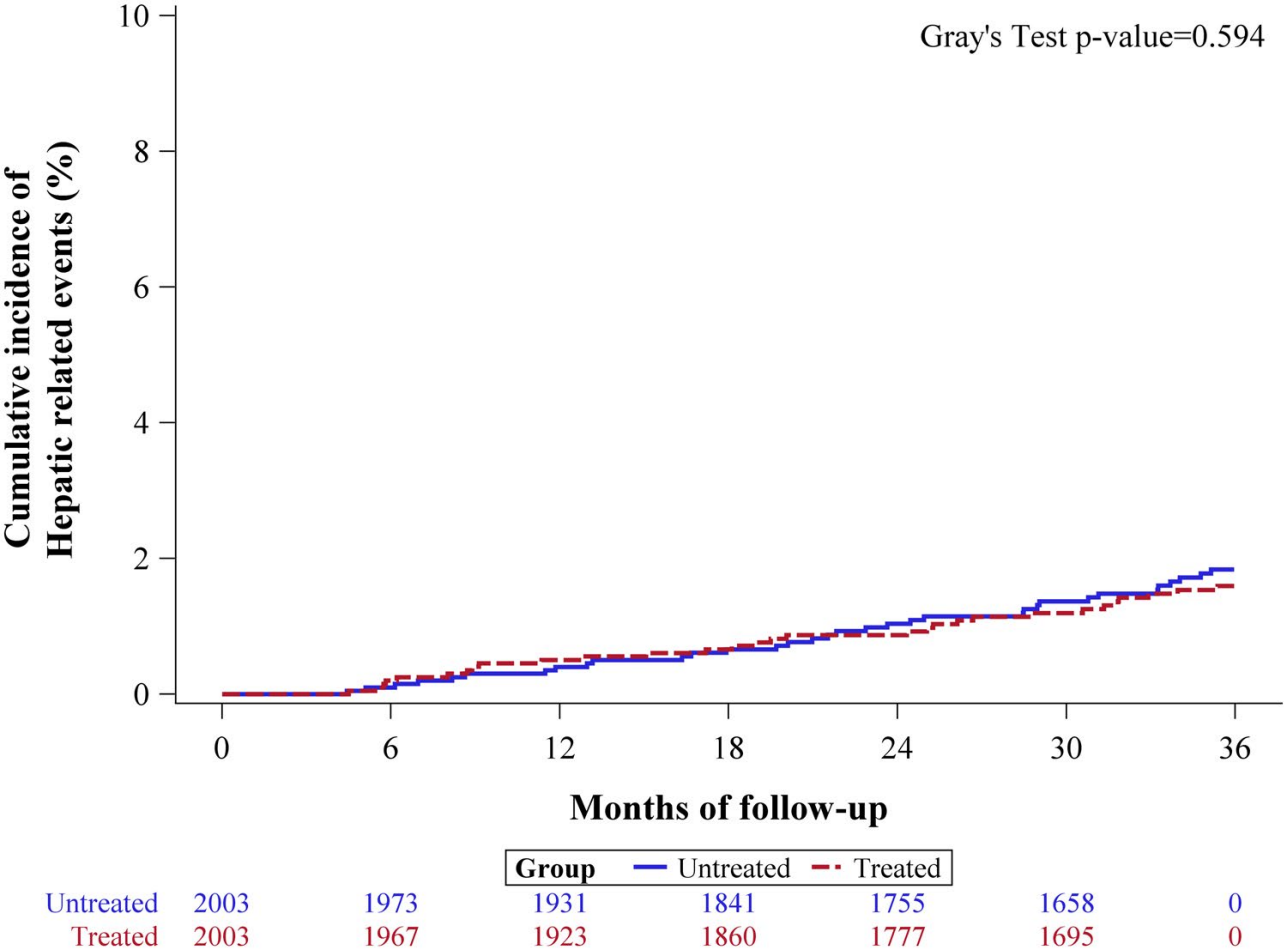
Cumulative Incidence Function (CIF) plot for liver-related events over a three-year follow-up period after adjustment for immortal time bias.

Similarly, for liver-related events, 30 patients (1.5%) in the aspirin group and 34 patients (1.7%) in the non-aspirin group developed liver-related complications over the three-year period. The competing risk analysis indicated that aspirin use did not significantly lower the risk of liver-related events, with an SHR of 0.88 (95% CI: 0.54–1.43, *p* = 0.596) compared to the non-aspirin cohort (Table 2 and Figure 3).

### Secondary outcomes

**Figure 4.**
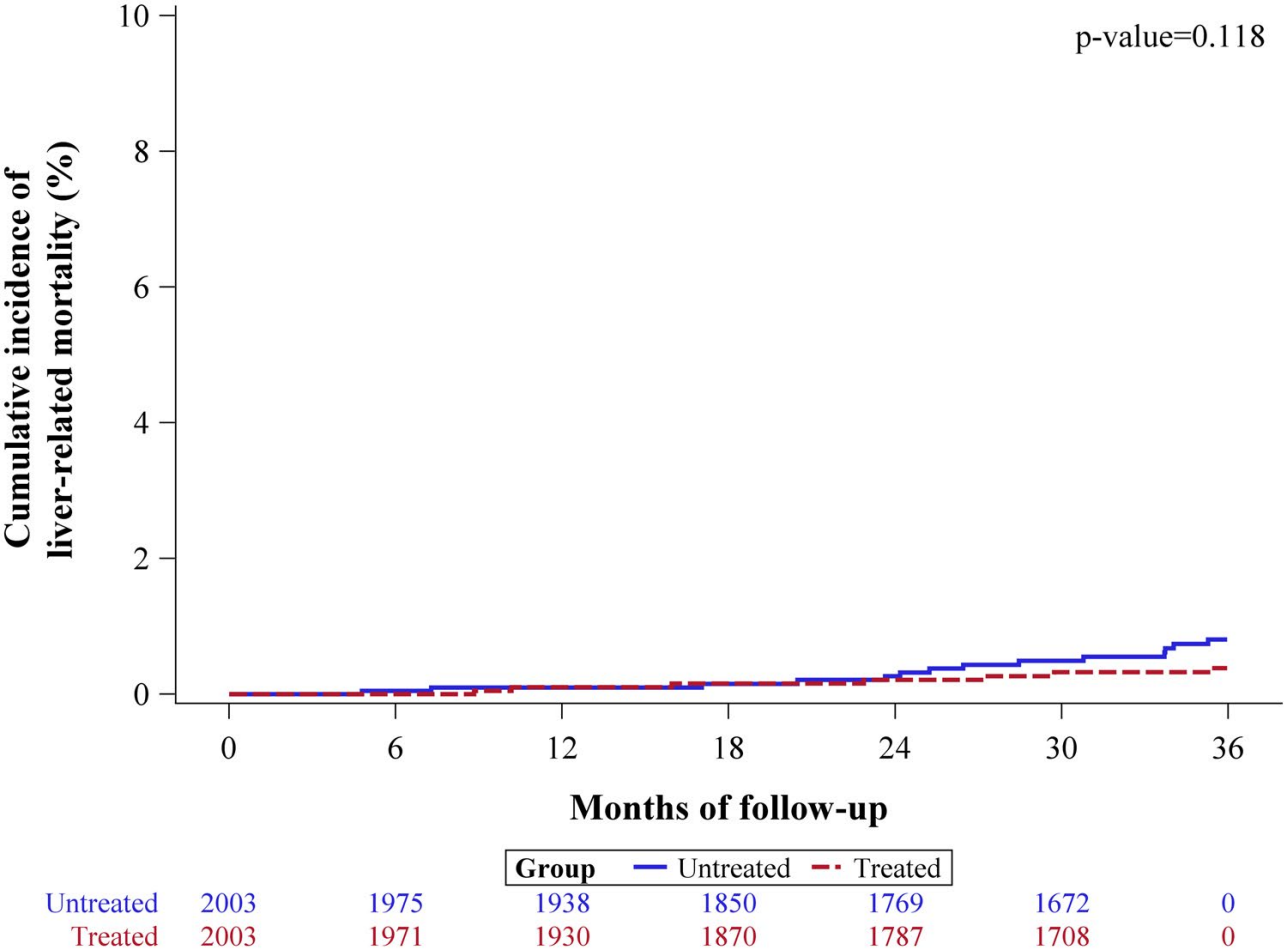
Cumulative Incidence Function (CIF) plot for liver-related mortality over a three-year follow-up period after adjustment for immortal time bias.

For secondary outcomes, including HCC incidence and liver-related mortality, no significant difference in the SHR was observed between the aspirin and non-aspirin cohorts over the follow-up period. Among 4,006 propensity score-matched patients (aspirin: 2,003; non-aspirin: 2,003):

Liver-related mortality occurred in 7 patients (0.3%) in the aspirin cohort and 14 patients (0.7%) in the non-aspirin cohort, with an SHR of 0.49 (95% CI: 0.20–1.22, *p* = 0.126) (Table 2 and Figure 4).

**Figure 5.**
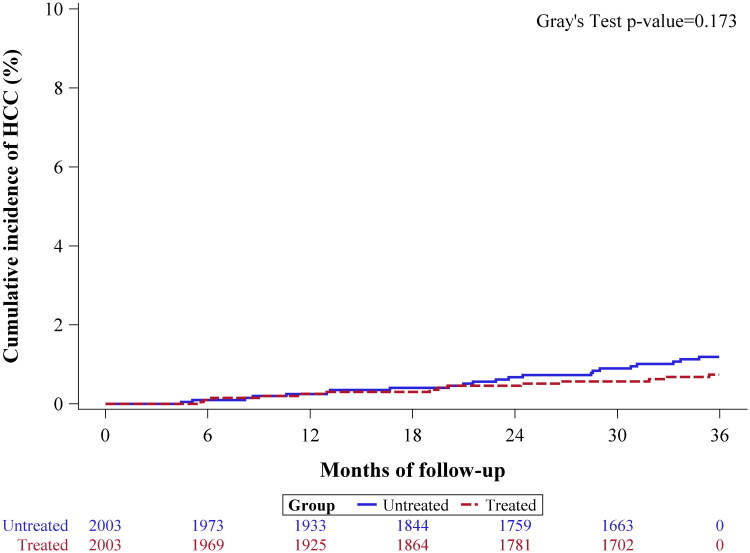
Cumulative Incidence Function (CIF) plot for HCC occurrence over a three-year follow-up period after adjustment for immortal time bias.

HCC incidence was observed in 14 patients (0.7%) in the aspirin cohort and 22 patients (1.1%) in the non-aspirin cohort, with a SHR of 0.63 (95% CI: 0.32–1.23, *p* = 0.178) (Table 2 and Figure 5).

### Major bleeding risk

**Figure 6.**
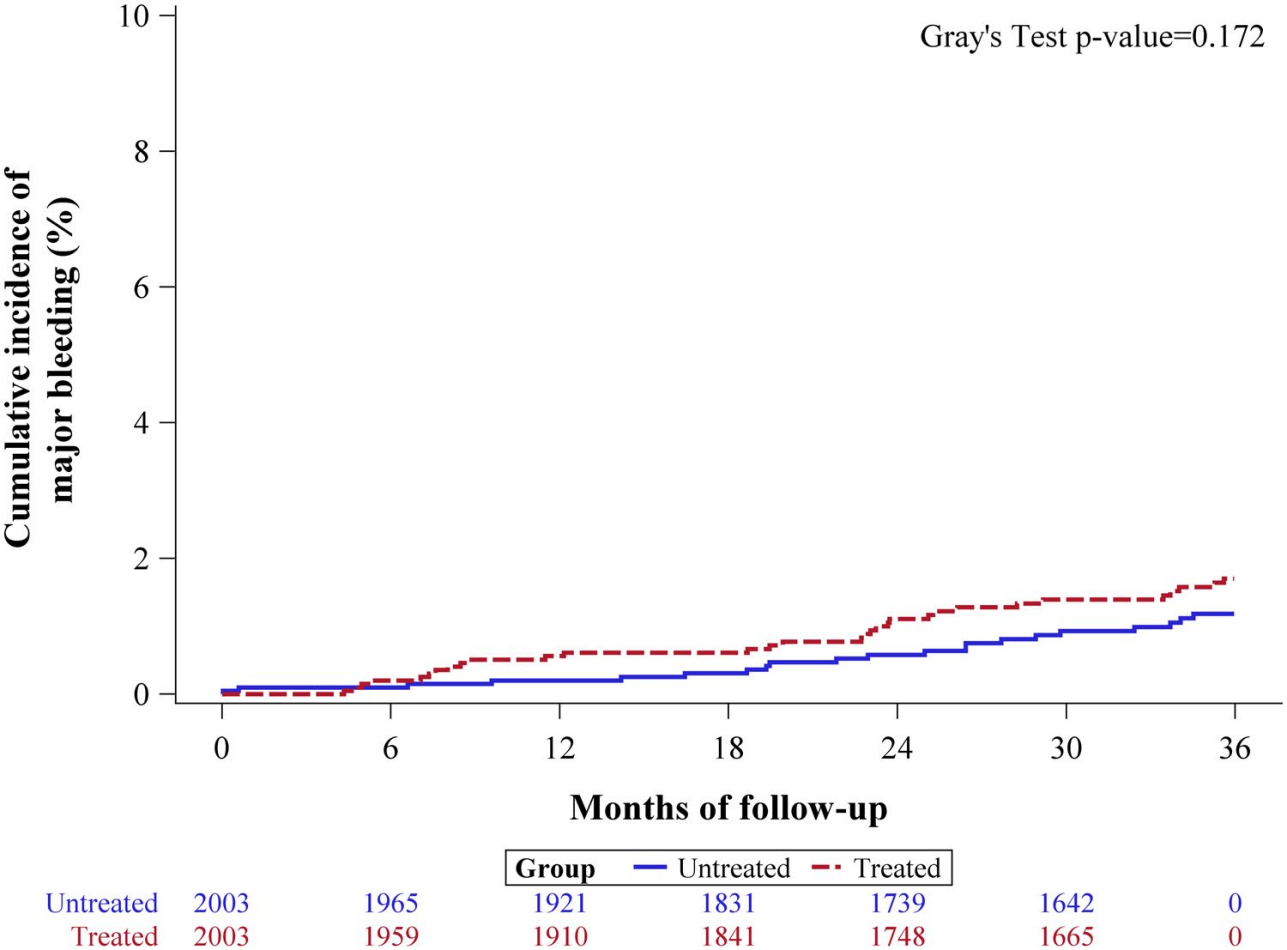
Cumulative Incidence Function (CIF) plot for major bleeding events over a three-year follow-up period after adjustment for immortal time bias. Supplementary Figure 1. Cumulative Incidence Function (CIF) plot for all-cause mortality in non-viral MASLD between the aspirin vs non-aspirin groups. Supplementary Figure 2. Cumulative Incidence Function (CIF) plot for liver-related events in non-viral MASLD between the aspirin vs non-aspirin groups. Supplementary Figure 3. Cumulative Incidence Function (CIF) plot for liver-related mortality in non-viral MASLD between the aspirin vs non-aspirin groups. Supplementary Figure 4. Cumulative Incidence Function (CIF) plot for HCC occurrence in non-viral MASLD between the aspirin vs non-aspirin groups. Supplementary Figure 5. Comparisons of the composite of CVD events between the aspirin vs non-aspirin groups. Supplementary Figure 6. Cumulative Incidence Function (CIF) plot for Liver-related events over a three-year follow-up period before adjustment for immortal time bias. Supplementary Figure 7. Cumulative Incidence Function (CIF) plot for HCC occurrence over a three-year follow-up period before adjustment for immortal time bias.

To evaluate the risk of major bleeding events associated with aspirin use, a competing risk analysis was conducted. As shown in Supplementary Table 4 and illustrated in Figure 6, aspirin use was not significantly associated with an increased incidence of major bleeding events (SHR: 1.47, 95% CI: 0.84–2.55, *p* = 0.175), as defined by ISTH criteria.

### Delta changes in steatosis and fibrosis scores

Subsequent analyses of the delta changes in steatosis and fibrosis scores—calculated as the difference between values at three years post-index date and baseline—revealed no significant reduction in the NAFLD-Liver Fat Score, Hepatic Steatosis Index (HSI), BARD score, AST to Platelet Ratio Index (APRI), and FIB-4 within the aspirin-treated cohort compared to the control cohort, as presented in Supplementary Table 5.

### A subgroup analysis focusing exclusively on patients with non-viral MASLD

To eliminate the potential confounding influence of viral hepatitis, a subgroup analysis was performed on patients with non-viral MASLD. As shown in Supplementary Figures 1–4, the aspirin-treated and untreated groups showed no statistically significant differences in all-cause mortality, liver-related events, HCC incidence, or liver-related mortality within this subgroup. These findings are consistent with the overall cohort results.

### Comparisons of composite of CVD events between the aspirin vs non-aspirin groups

Given the established association between metabolic dysfunction and cardiovascular risk [32], we examined the incidence of composite cardiovascular events as a secondary outcome. As shown in Supplementary Figure 5, the aspirin group exhibited a significantly higher cumulative incidence of cardiovascular events over the 3-year follow-up (Gray’s test *p* = 0.010).

### A preliminary analysis without correction for immortal time bias

In a preliminary analysis without correction for immortal time bias—but with baseline characteristics balanced through propensity score matching—aspirin use was significantly associated with lower liver-related events (SHR = 0.42, 95% CI: 0.24–0.73, *p* = 0.002; Supplementary Figure 6) and HCC incidence (SHR = 0.30, 95% CI: 0.13–0.70; Supplementary Figure 7) over a three-year follow-up. However, these associations were attenuated after accounting for immortal time bias through index date matching and adjustment, highlighting the importance of appropriate methodological approaches in estimating the true effect of aspirin.

## Discussion

Our propensity-matched cohort study, employing stringent exclusion criteria (e.g. outcome events within three months post-index, alcoholic liver disease, six major causes of cardiovascular death unrelated to the pre-defined composite CVD events, and concurrent use of other antiplatelet agents), was further strengthened by adjustments for immortal time bias. Utilizing a large multi-institutional database and competing risk strategies, we found that low-dose aspirin alone was not significantly associated with reduced all-cause mortality, liver-related events, liver-related mortality, or HCC incidence in MASLD patients over a three-year follow-up. Similarly, no significant improvements in steatosis or fibrosis scores were observed, consistent with the overall outcome findings. However, a trend toward lower liver-related mortality, HCC incidence, and HSI improvement was noted without an increased risk of major bleeding. While these trends did not reach statistical significance, they suggest a potential benefit warranting further exploration. These findings challenge prior studies, highlighting the need for further investigation into aspirin’s role, particularly in combination with synergistic therapies, through randomized clinical trials exceeding three years.

Our findings contrast with previous studies that have suggested a beneficial role of aspirin in reducing liver-related events and HCC incidence in chronic liver disease. However, much of the supporting evidence comes from patients with chronic viral hepatitis. Prior studies have consistently shown that low-dose aspirin use in chronic viral hepatitis patients correlates with a significantly reduced incidence of HCC and lower liver-related mortality [12–15,33], without a corresponding increase in gastrointestinal bleeding risk [14]. Systematic review and meta-analysis have further corroborated the association of aspirin with a decreased incidence of HCC in patients with cirrhosis and virus hepatitis [34–36]. In contrast, the long-term effects of daily aspirin use in MASLD remain poorly defined, particularly regarding all-cause mortality and liver-related complications [18]. Preliminary evidence suggests that antiplatelet therapy, including aspirin, might attenuate fibrosis progression in biopsy-proven NAFLD cases [16,17] and potentially decrease the risk of NAFLD-related HCC [16,18]. A recent randomized clinical trial reported that six months of daily low-dose aspirin substantially reduced hepatic fat content in MASLD patients compared to placebo [20]. The discrepancies between our findings and previous studies may stem from differences in study design and patient selection. Unlike prior studies that often included broader cardiovascular profiles and allowed concurrent antiplatelet use, our study employed strict inclusion criteria to refine patient and medication classification. Beyond ICD codes [19], we incorporate BMI, blood pressure, and laboratory data to ensure a more metabolically homogeneous MASLD cohort. Additionally, while MASLD allows for the coexistence of other liver diseases (e.g. MASLD with autoimmune hepatitis or viral hepatitis [1], many previous MASLD studies excluded viral hepatitis or autoimmune hepatitis, potentially contributing to outcome variations.

Another possible explanation for the non-significant findings regarding aspirin’s effect on reducing overall mortality and liver-related events, including HCC risk, is the relatively short three-year follow-up period. Prior evidence suggests that the protective effect of aspirin may be duration-dependent [19], with the greatest benefit observed after four or more years of use [17]. One study reported a significantly lower HCC risk in individuals using aspirin for ≥3 years compared to those with short-term use (<1 year) [18]. However, achieving long-term adherence to aspirin therapy in clinical trials remains challenging, particularly in individuals without cardiovascular indications. Additionally, several prior studies that demonstrated positive outcomes allowed concurrent use of other antiplatelet agents in the aspirin-treated group, raising the possibility that aspirin alone may be insufficient to achieve these benefits. This suggests that dual-antiplatelet therapy could provide synergistic effects. Mechanistically, aspirin irreversibly inhibits cyclooxygenase-1 (COX-1), thereby suppressing thromboxane A2-mediated platelet aggregation, whereas P2Y12 inhibitors such as clopidogrel inhibit ADP-induced platelet activation. This dual antiplatelet blockade may enhance antithrombotic activity [37] and reduce microthrombi formation or sinusoidal thrombosis, potentially mitigating portal hypertension or hepatic ischemia-related injury [38,39]. Nevertheless, this hypothesis remains speculative, and mechanistic validation through experimental or translational studies is warranted. Finally, although not statistically significant, the numerically lower liver-related mortality and HCC incidence, along with the greater improvement in HSI observed in the aspirin-treated group (−0.52 ± 5.02 vs. −0.26 ± 4.66; *p* = 0.215), may suggest a potential protective signal in our study. These findings should be interpreted with caution due to limited statistical power but underscore the need for further prospective studies with larger sample sizes, extended follow-up (≥4 years), and evaluation of combination strategies with synergistic agents to clarify aspirin’s therapeutic role in MASLD.

After accounting for immortal time bias, our current results differ from our prior analyses that did not adjust for this bias, which had previously indicated a protective effect of aspirin on HCC and liver-related events over a three-year follow-up (Supplementary Figures 6 and 7). To mitigate immortal time bias, we matched index dates and balanced baseline clinical characteristics (Figure 1), an approach recognized as an effective method to reduce this bias and ensure comparability between groups [40]. This strategy ensures that both groups begin from a common time point with similar health profiles, thereby enhancing the validity and reliability of our findings.

We observed no significant increase in major bleeding events in the aspirin-treated group compared to the untreated group. This finding aligns with a previous study that reported aspirin therapy did not carry an augmented risk of acute variceal hemorrhage, gastrointestinal bleeding, or worsening anemia in cirrhotic patients undergoing evaluation for liver transplantation [41]. Despite this reassuring safety profile, aspirin remains underutilized in the management of coronary artery disease (CAD) in decompensated cirrhotic patients [41]. Furthermore, although not statistically significant, the numerically higher incidence of major bleeding in the aspirin group (1.54% vs. 1.05%, *p* = 0.175) suggests a potential safety signal. Notably, the aspirin group exhibited a significantly higher cumulative incidence of cardiovascular events over the 3-year follow-up. This counterintuitive result may reflect confounding by indication, as patients prescribed aspirin likely had a higher baseline cardiovascular risk that may not have been fully accounted for despite PSM. This observation aligns with prior studies in older populations where aspirin did not provide a net cardiovascular benefit and increased bleeding risk [42]. These findings should be interpreted with caution and warrant further validation in larger prospective studies with granular cardiovascular risk stratification.

One limitation of our study is its retrospective design, lacking prospective validation or cross-national replication. Nevertheless, our findings provide a solid foundation for future clinical trials. Over a three-year follow-up, aspirin use alone did not significantly impact the primary outcome or improve steatosis and fibrosis scores, suggesting that its benefits may be limited without adjunctive therapies, lifestyle modifications, or prolonged use (≥4 years). Another limitation is that we did not apply a time-varying model for outcome analysis. Although time-varying exposure modeling is ideal for accounting for fluctuations in aspirin use, it introduces substantial complexity in large datasets, requiring frequent updates and detailed exposure tracking. Given that aspirin use remained relatively stable in our cohort, we opted for propensity score matching (PSM) with baseline characteristic balancing and matched index dates, a validated method to mitigate immortal time bias [40]. Additionally, unmeasured confounders such as smoking and dietary factors were not considered, which may have influenced our results. Furthermore, the relatively small number of events limited statistical power, though we mitigated this by employing rigorous statistical methods and controlling for potential confounders, as demonstrated in systematic reviews comparing lean and non-lean NAFLD patients [43]. Furthermore, our study included MASLD patients with concurrent viral hepatitis, reflecting the real-world overlap between metabolic dysfunction and chronic liver disease. While previous NAFLD studies often excluded viral hepatitis, we conducted sensitivity analyses excluding viral hepatitis cases, confirming consistent findings and strengthening our conclusions regarding aspirin’s association with mortality and liver-related events. Another limitation is phenotype ascertainment: MASLD was defined using NAFLD ICD-9/10 codes plus ultrasound or CT/MRI evidence of steatosis, and alcohol-related liver disease was excluded by ICD-10 and ICD-9 codes. However, the CGRD lacks structured daily alcohol-intake data, preventing application of at-risk thresholds and precluding MetALD/ALD subgroup analyses. Consequently, some MetALD/ALD misclassification within the MASLD cohort cannot be excluded; because alcohol-related phenotypes carry higher liver-event risks, any contamination would likely bias liver-related estimates away from the null [1,44]. Accordingly, our inferences apply to ‘pure’ MASLD rather than ALD/MetALD. For baseline variables, most data were complete; noninvasive tests (e.g. APRI, FIB-4, MELD) were available only in subsets. We performed complete-case analyses without imputation and did not include these NITs in the PSM; balance between the two groups was adequate. Finally, CVD remains a major competing risk in MASLD patients, potentially attenuating aspirin’s hepatoprotective effects [45]. While aspirin may offer hepatic benefits, its cardioprotective role complicates interpretation of its effect on liver-related outcomes. This suggests that reductions in liver-related mortality may be overshadowed by concurrent cardiovascular mortality, particularly in high-risk individuals. Future studies with longer follow-up and stratified cardiovascular risk assessments are essential to clarify aspirin’s long-term impact on MASLD progression.

In conclusion, this large, multi-institutional cohort study found no significant association between aspirin use and reduced all-cause mortality, liver-related events, liver-related mortality, or HCC incidence in MASLD patients over a three-year follow-up. Notably, aspirin use alone did not increase the risk of major bleeding events. However, a downward trend in liver-related mortality, HCC incidence, and HSI improvement was observed. While these trends did not reach statistical significance, they suggest a potential long-term benefit warranting further investigation. These findings challenge prior studies that have reported a protective effect of aspirin on liver-related outcomes, emphasizing the need for randomized clinical trials exceeding three years to assess its potential role, particularly in combination with synergistic therapies. Furthermore, our results provide clinical guidance, suggesting that routine aspirin use may not be warranted for MASLD patients without cardiovascular indications. Future research should explore longer treatment durations and combination strategies to determine aspirin’s definitive role in MASLD management.

## Supplementary Material

Supplemental Material

## Data Availability

Data are not publicly available but may be accessed upon reasonable request, subject to institutional review board (IRB) approval and in accordance with our institutional data sharing policy. Requests should be directed to Prof. Shang-Hung Chang, M.D., Ph.D. E-mail: afen.chang@gmail.com.
