# Supplementary material for: Association of aspirin use alone with mortality and liver-related events in MASLD: a multi-institutional three-year study
Source: Ann Med. 2025 Oct 17;57(1):2573146. doi: 10.1080/07853890.2025.2573146 (PMC12536622; doi:10.1080/07853890.2025.2573146)
Supplement: Supplemental Material [file IANN_A_2573146_SM6362.zip › suppl_data/20250618 Supplementary Table 1 to 5, Fig caption.docx]

**Supplementary Table 1.** Definitions of alcoholic liver disease and six major causes of cardiovascular death unrelated to the pre-defined composite CVD events^1^

| Outcomes | ICD-9 codes | ICD-10 codes |
| --- | --- | --- |
| Alcoholic liver disease | 5710, 5711, 5712, 5713 | K70 |
| Six causes of cardiac death unrelated to composite CVD events |  |  |
| Aortic dissection | 441 | I71 |
| Pericarditis | 420, 423 | I30, I31, I32 |
| Endocarditis | 421, 42490, 42491, 42499 | I33, I38, I39 |
| Myocarditis | 422, 429 | I40, I41 |
| Rheumatic heart disease | 390-398, | I01, I02, I05-I09 |
| Pulmonary hypertension | 4160 | I27 |

Cardiovascular diseases (CVDs)

| ^1^Almuwaqqat Z, Hui Q, Liu C, et al. Long-Term Body Mass Index Variability and Adverse Cardiovascular Outcomes. JAMA Netw Open 2024; 7(3): e243062. |
| --- |
|  |
|  |

**Supplementary Table 2.** The definition of primary outcomes: The occurrence of HCC and liver-related events after the index date respectively.

| Outcomes | ICD-9 codes | ICD-10 codes |
| --- | --- | --- |
| All-cause mortality | Causes of Death Statistics Data File | |
| Liver-related events |  |  |
| Esophageal variceal bleeding | 456.0, 456.2 | I85.01, I85.11 |
| Ascites | 789.5, 568.82 | R18.8, K70.31 |
| Hepatic encephalopathy | 572.2 | K72.91 |
| Peritonitis | 56723, 56729 | K65.2 |
| Liver transplantation | 99682;  PCS: 505 | Z944, V427, T864;  PCS: 0FY00Z0, 0FY00Z1, 0FY00Z2 |
| Hepatorenal syndrome | 5724 | K767 |

**Supplementary Table 3.** The definition of secondary outcomes: The occurrence of liver-related mortality, all-cause mortality and major bleeding events after the index date respectively

| Outcomes | ICD-9 codes | ICD-10 codes |
| --- | --- | --- |
| HCC | 1550 | C220, C228 |
| Liver-related mortality | Causes of Death Statistics Data File attributed to the first five diagnostic ICD code: 571, 456, 570, 572, 573, 070, 155 | K70, K73, K74, I85, K71, K72, K75, K76, B15, B16, B17, B18, B19, C22 |
| Major bleeding events |  |  |
| Intracranial bleeding | 430, 431, 432.0, 432.9 | I60, I61, I62.0, I62.1, I62.9 |
| Gastrointestinal bleeding  (GI bleeding) | 531.0, 531.2, 531.4, 531.6, 532.0, 532.2, 532.4, 532.6, 533.0, 533.2, 533.4, 533.6, 534.0, 534.2, 534.4, 534.6, 569.3, 578.0, 578.1, 578.9 | K92.0, K92.1, I85.0, I98.20, I98.3, K22.10, K22.12, K22.14, K22.16, K25.0, K25.2, K25.4, K25.6, K26.0, K26.2, K26.4, K26.6, K27.0, K27.2, K27.4, K27.6, K28.0, K28.2, K28.4, K28.6, K29.0, K63.80, K31.80, K55.20, K62.5, K92.2. |
| Other critical site bleeding | 287.8, 287.9, 596.7, 784.8, 599.7, 627.1, 459.0, 719.1, 786.3 | N02.0, N02.1, N02.2, N02.3, N02.4, N02.5, N02.6, N02.7, N02.8, N02.9, K66.1, N93.8, N93.9, N95.0, R04.1, R04.2, R04.8, R04.9, R31.0, R31.1, R31.8, R58, D68.3, H35.6, H43.1, H45.0, M25.0 |

**Supplementary Table 4.** Competing risk analysis for the major bleeding events over 3 years of follow-up.

|  | N | Events | SHR | 95%CI | p-value | Competing risk |
| --- | --- | --- | --- | --- | --- | --- |
| Aspirin | 2003 | 31 | 1.47 | (0.84-2.55) | 0.175 | all-cause mortality |
| Non-aspirin | 2003 | 21 |  |  |  |  |

N, number of patients; SHR, subdistribution hazard ratio; CI, confidence interval

**Supplementary Table 5.** Comparisons of the various steatosis and fibrosis scores delta change (values after 3 years minus values at index date) between the case and control cohort.

| Variable (n=number of patients with available delta values) | **Case  (Aspirin-treated)  (*n* = 2003)** | **Control  (No aspirin-treated) (*n* =2003)** | p-value |
| --- | --- | --- | --- |
| NAFLD-Liver Fat Score (120) | -0.68 (-1.32~0.46) | -0.53 (-2.26~0.72) | 0.643 |
| Hepatic steatosis index (HSI) (987) | -0.52±5.02 | -0.26±4.66 | 0.215 |
| BARD score (987) | 0.19±1.14 | 0.12±1.13 | 0.259 |
| APRI (168) | -0.08 (-0.23~-0.02) | -0.05 (-0.49~0.14) | 0.951 |
| FIB-4 index (165) | -0.25 (-0.84~0.15) | 0.06 (-0.62~0.49) | 0.389 |

HSI: Hepatic Steatosis Index; APRI: AST to Platelet Ratio Index; FIB-4

: Fibrosis-4

**Supplementary Figure 1.** Cumulative Incidence Function (CIF) plot for all-cause mortality in non-viral MASLD between the aspirin vs non-aspirin groups.
**Supplementary Figure 2.** Cumulative Incidence Function (CIF) plot for liver-related events in non-viral MASLD between the aspirin vs non-aspirin groups.
**Supplementary Figure 3.** Cumulative Incidence Function (CIF) plot for liver-related mortality in non-viral MASLD between the aspirin vs non-aspirin groups.
**Supplementary Figure 4.** Cumulative Incidence Function (CIF) plot for HCC occurrence in non-viral MASLD between the aspirin vs non-aspirin groups.
**Supplementary Figure 5.** Comparisons of the composite of CVD events between the aspirin vs non-aspirin groups.
**Supplementary Figure 6.** Cumulative Incidence Function (CIF) plot for Liver-related events over a three-year follow-up period before adjustment for immortal time bias.
**Supplementary Figure 7.** Cumulative Incidence Function (CIF) plot for HCC occurrence over a three-year follow-up period before adjustment for immortal time

bias
