# Supplementary material for: Association of aspirin use alone with mortality and liver-related events in MASLD: a multi-institutional three-year study
Source: Ann Med. 2025 Oct 17;57(1):2573146. doi: 10.1080/07853890.2025.2573146 (PMC12536622; doi:10.1080/07853890.2025.2573146)
Supplement: Supplemental Material [file IANN_A_2573146_SM6362.zip › suppl_data/Supplementary Figure 1 All cause mortality in non viral MASLD between the aspirin vs non aspirin group copy.pdf]

Supplementary Figure 1.

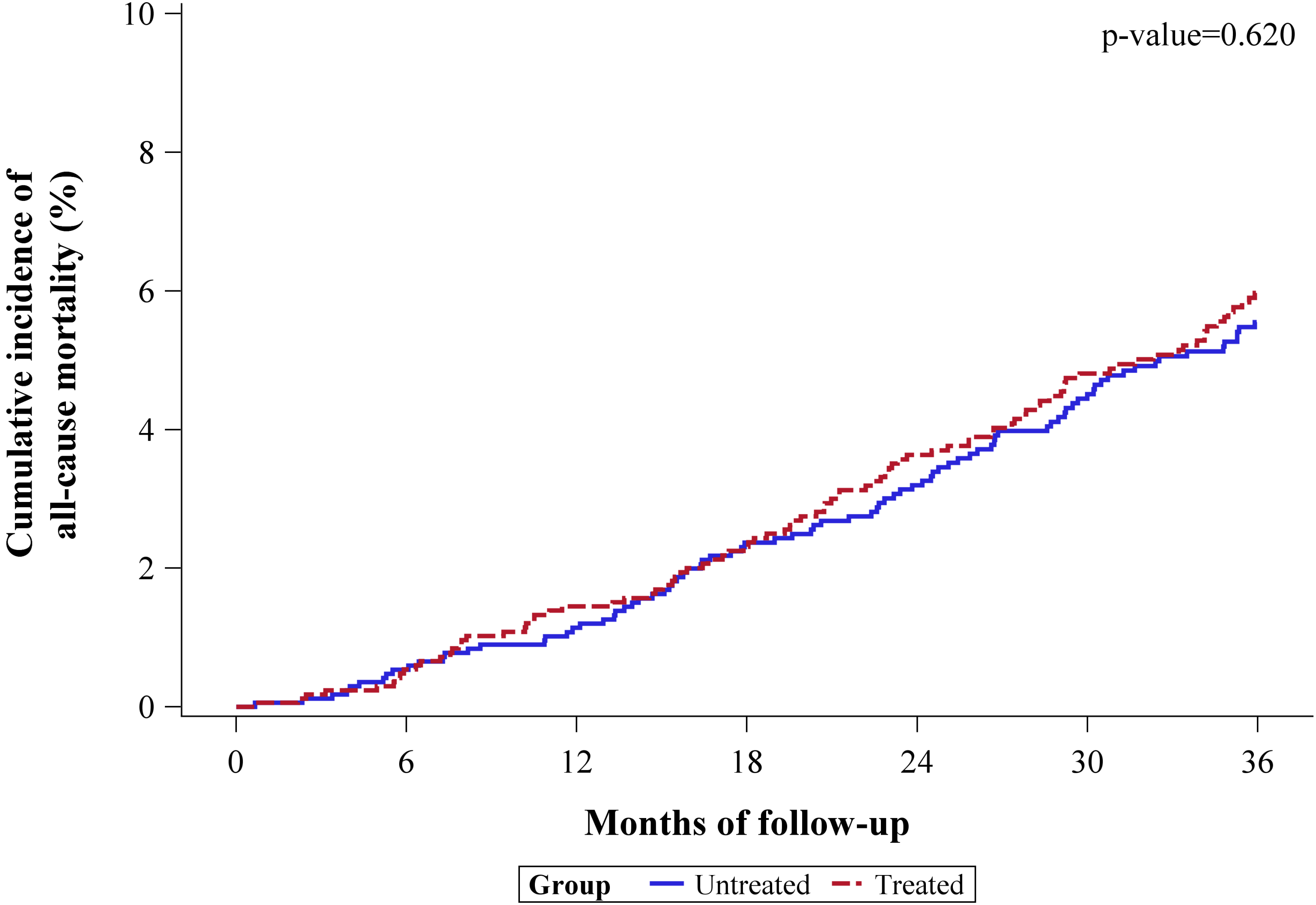

	0	6	12	18	24	30	36
Untreated	1678	1659	1633	1561	1500	1422	0
Treated	1678	1649	1616	1571	1504	1439	0
