# Supplementary material for: Association of aspirin use alone with mortality and liver-related events in MASLD: a multi-institutional three-year study
Source: Ann Med. 2025 Oct 17;57(1):2573146. doi: 10.1080/07853890.2025.2573146 (PMC12536622; doi:10.1080/07853890.2025.2573146)
Supplement: Supplemental Material [file IANN_A_2573146_SM6362.zip › suppl_data/Supplementary Figure 5 Comparisons of composite of CVD events between the aspirin vs non aspirin group copy.pdf]

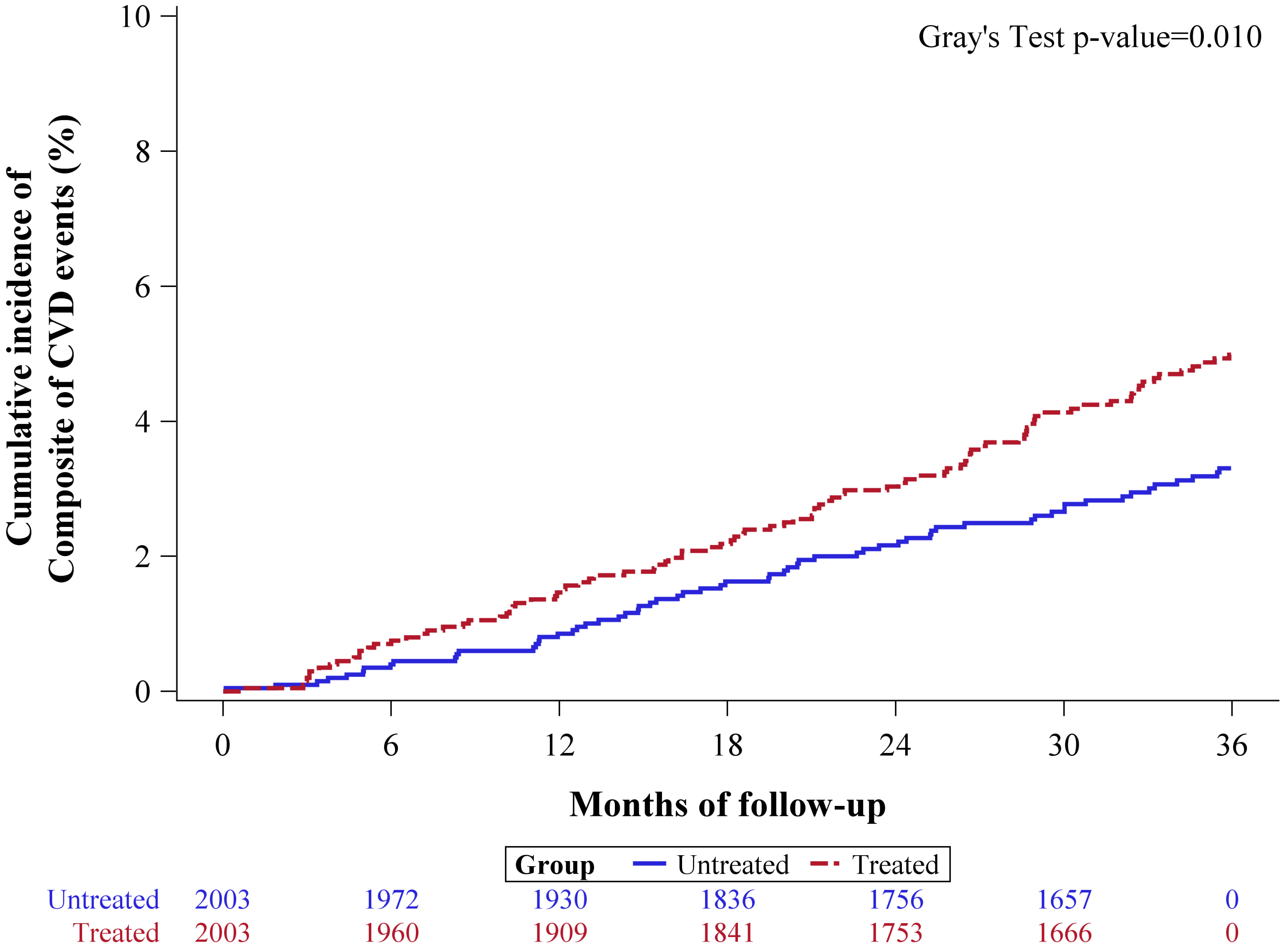
Gray's Test p-value=0.010
Cumulative incidence of
Composite of CVD events (%)
0
2
4
6
8
10
0
6
12
18
24
30
36
Months of follow-up
Group
Untreated
Treated
Untreated 2003 1972 1930 1836 1756 1657 0
Treated 2003 1960 1909 1841 1753 1666 0
