# Supplementary material for: Association of aspirin use alone with mortality and liver-related events in MASLD: a multi-institutional three-year study
Source: Ann Med. 2025 Oct 17;57(1):2573146. doi: 10.1080/07853890.2025.2573146 (PMC12536622; doi:10.1080/07853890.2025.2573146)

Supplementary Figure 6.

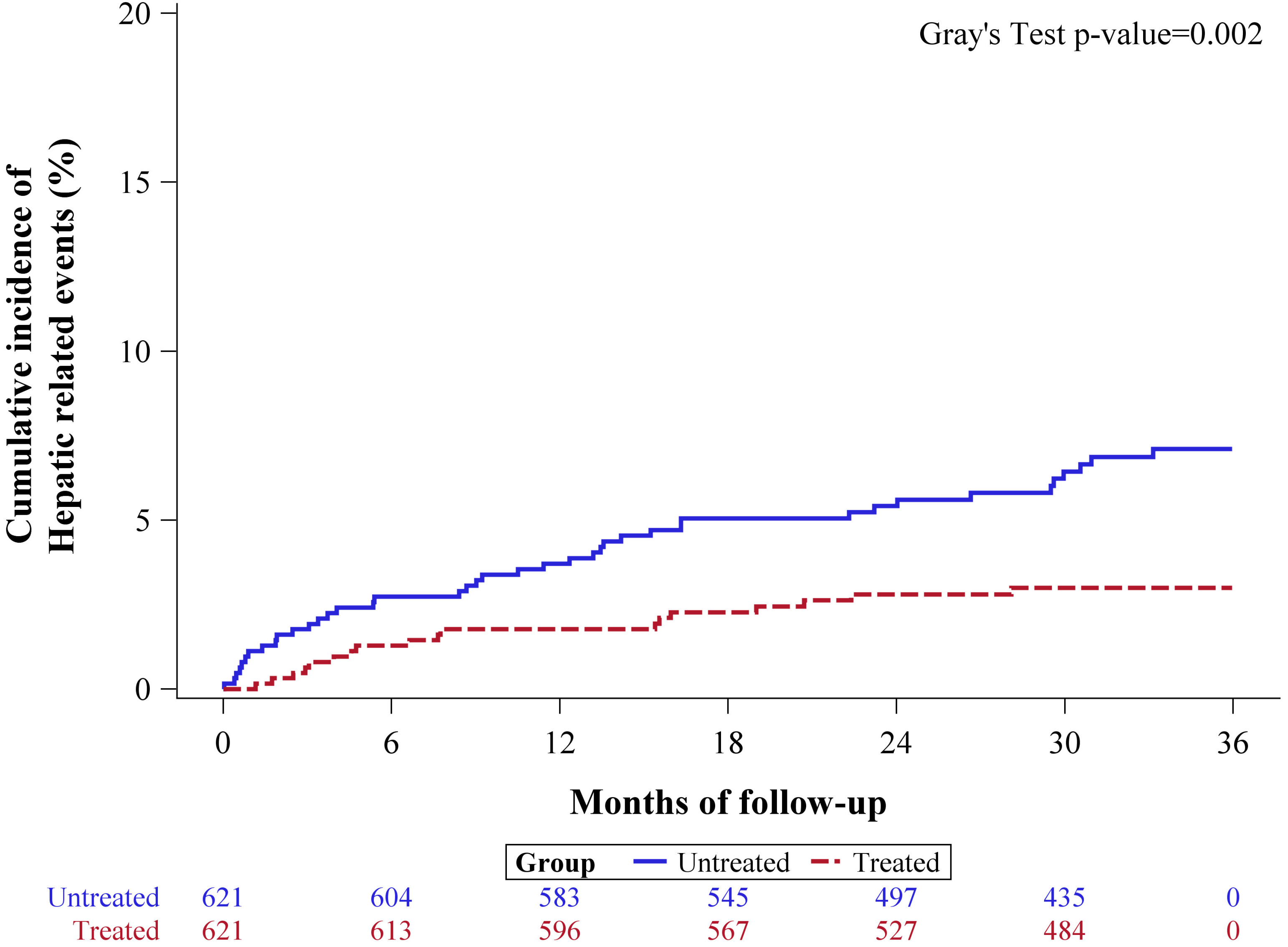

Supplement: Supplemental Material [file IANN_A_2573146_SM6362.zip › suppl_data/Supplementary Figure 6 Hepatic related events brefore adjustment for immortal time biase copy.pdf]
