# Supplementary figures and images for: Association of aspirin use alone with mortality and liver-related events in MASLD: a multi-institutional three-year study
Source: Ann Med. 2025 Oct 17;57(1):2573146. doi: 10.1080/07853890.2025.2573146 (PMC12536622; doi:10.1080/07853890.2025.2573146)

**Cumulative incidence of liver-related mortality (%)**

## Months of follow-up

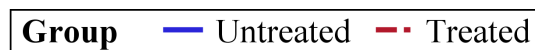

|           |      |      |      |      |      |      |   |
|-----------|------|------|------|------|------|------|---|
| Untreated | 1678 | 1659 | 1633 | 1561 | 1499 | 1422 | 0 |
| Treated   | 1678 | 1649 | 1616 | 1571 | 1504 | 1439 | 0 |

Supplement: Supplemental Material [file IANN_A_2573146_SM6362.zip › suppl_data/Supplementary Figure 3 Liver related mortality in non viral MASLD between the aspirin vs non aspirin group copy.pdf]

Supplementary Figure 4.

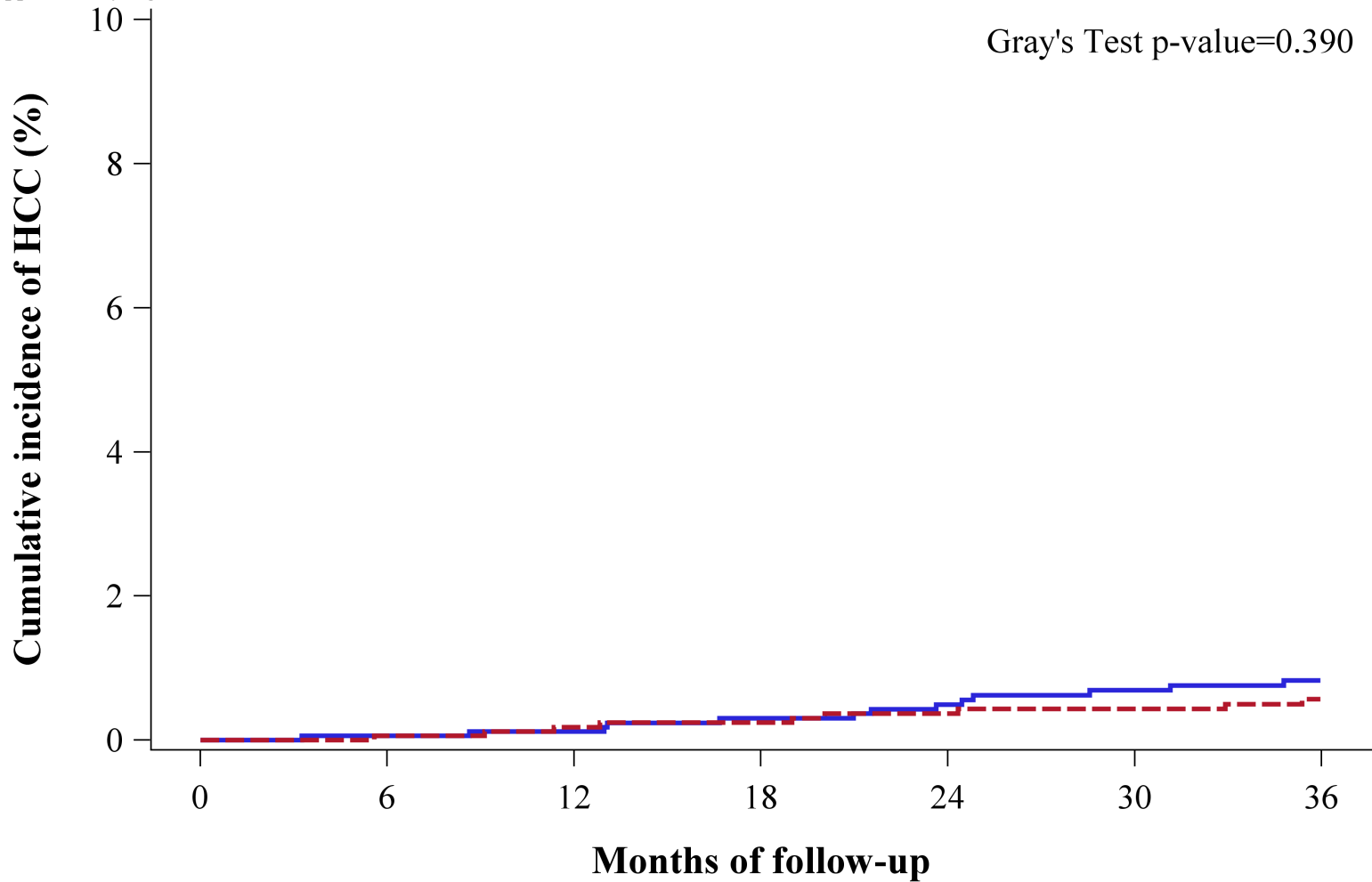

|           |      |      |      |      |      |      |   |
|-----------|------|------|------|------|------|------|---|
| Untreated | 1678 | 1658 | 1631 | 1556 | 1492 | 1415 | 0 |
| Treated   | 1678 | 1648 | 1613 | 1567 | 1499 | 1434 | 0 |

Supplement: Supplemental Material [file IANN_A_2573146_SM6362.zip › suppl_data/Supplementary Figure 4 HCC incidence in non viral MASLD between the aspirin vs non aspirin group copy.pdf]

Supplementary Figure 7.

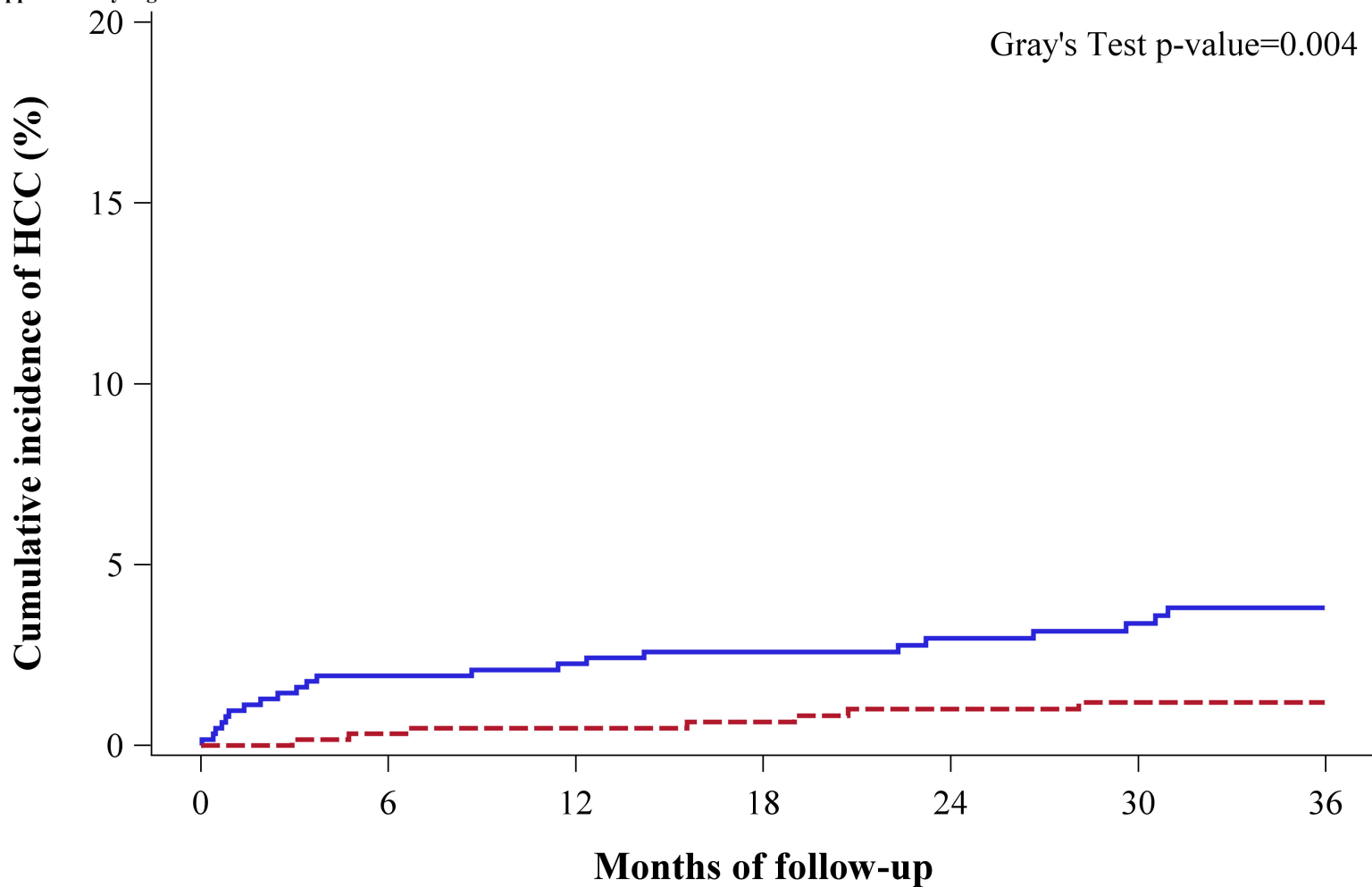

|           |     |     |     |     |     |     |   |
|-----------|-----|-----|-----|-----|-----|-----|---|
| Untreated | 621 | 609 | 591 | 553 | 504 | 444 | 0 |
| Treated   | 621 | 619 | 603 | 573 | 532 | 488 | 0 |

Supplement: Supplemental Material [file IANN_A_2573146_SM6362.zip › suppl_data/Supplementary Figure 7 HCC incidence brefore adjustment for immortal time biase copy.pdf]
